# Water status diagnosis in greenhouse drip-irrigated tomato and celery using leaf turgor dynamics and machine learning

**DOI:** 10.3389/fpls.2025.1743809

**Published:** 2026-01-16

**Authors:** Quanyue Xu, Ruixia Chen, Xufeng Li, Hongxiang Wu, Juanjuan Ma, Lijian Zheng

**Affiliations:** 1 College of Water Resource Science and Engineering, Taiyuan University of Technology, Taiyuan, China; 2 Shanxi Key Laboratory of Collaborative Utilization of River Basin Water Resources, Taiyuan University of Technology, Taiyuan, China

**Keywords:** greenhouse crops, leaf turgor pressure, plant water status, precision irrigation, Random Forest

## Abstract

**Introduction:**

Accurate crop water status monitoring is crucial for optimized irrigation in controlled environments, but traditional approaches relying on damaging measurements or sporadic sampling frequently restrict real-time evaluation.

**Methods:**

This study explored the non-invasive leaf patch clamp pressure (LPCP) probe to evaluate the water status of drip-irrigated tomato and celery. Leaf turgor dynamics analysis enabled the characterization of the LPCP probe’s output parameter (P_p_) and its environmental drivers, and the development of predictive machine learning models.

**Results:**

The results indicated that diurnal patterns of P_p_ in drip-irrigated tomato and celery exhibited two distinct states: State I (unimodal) and State II (troughed), corresponding to moisture conditions with no or mild stress, and severe stress, respectively. The soil water content (SWC) thresholds for State I were set at SWC > 20% (tomato) and SWC > 19% (celery), whereas those for State II were set at SWC < 18% (tomato) and SWC < 16% (celery). For State I, P_p_ was positively associated with solar radiation but negatively associated with SWC (in tomato) and wind speed (in celery). For State II, the associations between P_p_ and environmental parameters were less than those in State I. Interestingly, compared to full irrigation, non-full irrigation treatments not only showed a higher proportion of State II but also resulted in an increase in both P_p,max_ and P_p,min_ by 15.39%–138.39% in tomato and 3.44%–94.02% in celery. These analytical results yielded four model parameter combinations based on the inclusion of SWC and the management of distinct P_p_ states. The prediction model that integrated Combination 4 (substate P_p_ prediction based on meteorological factors and SWC) with the random forest approach exhibited the highest accuracy (R_2_ = 0.995, MSE = 2.419, RMSE = 1.540, and MAE = 0.531), with SWC identified as its key feature parameter.

**Discussion:**

These findings provide a scientific foundation for optimizing the precision irrigation of greenhouse vegetables in drip systems.

## 1 Introduction

Water scarcity poses a significant bottleneck for irrigated greenhouse crop production, necessitating precise water status sensing for sustainable farming (Dutta et al., 2025). Tomato and celery, as representative greenhouse crops with growing demand (Gao et al., 2025; Martelli et al., 2024), are particularly constrained by their substantial water requirements. Consequently, this significant irrigation requirement has hindered production expansion in the context of limited water resources. Meanwhile, the varied water requirements of tomato and celery at different development stages complicate irrigation management (Huang et al., 2023; Zhang et al., 2024). Therefore, it is essential to precisely monitor the water status of these crops and optimize irrigation strategies to effectively address the aforementioned irrigation difficulties.

Traditional physiological indicators of plant water status (e.g., stomatal conductance, leaf water potential) are sampling-destructive and preclude continuous automated monitoring, failing to meet the continuity requirements of precise irrigation diagnosis (Fernández et al., 2017). Leaf turgor pressure, defined as the protoplast’s pressure against the cell wall, directly indicates plant water balance (Blackman, 2018). The leaf patch clamp pressure (LPCP) probes offer a nondestructive solution for long-term, continuous, *in situ* monitoring of leaf turgor. The LPCP probes’ output parameter (P_p_) exhibits an inverse relationship with leaf turgor pressure, meaning a higher P_p_ signifies lower turgor pressure in the leaf (Zimmermann et al., 2008). Compared with established physiological indicators, P_p_ offers enhanced feedback on plant water dynamics and allows for more accurate quantification of water demand and consumption characteristics throughout the development stages (Camoglu et al., 2021; Chen et al., 2024; Li et al., 2022; Zimmermann et al., 2013). For instance, Lee et al. (2012) utilized LPCP probes to initially explore the diurnal patterns of leaf turgor pressure in potted tomatoes, but the diurnal variation of P_p_ in tomatoes and celery under fluctuating soil moisture conditions requires further investigation. While P_p_ diurnal patterns for diagnosing plant water status have been well-established and exemplified by their application to irrigation decisions in commercial olive orchards (Padilla-Diaz et al., 2016; 2018), it remains uncertain whether this strategy is transferable to greenhouse tomato and celery. Therefore, it is also crucial to obtain sufficient data on daily P_p_ fluctuations for these crops and conduct in-depth analysis in conjunction with varying irrigation conditions, in order to accurately identify water stress thresholds.

Considering the limitations of direct and continuous sensing, along with the economic considerations for large-scale deployment, the development of predictive models for leaf turgor pressure is crucial. Many researchers have attempted to develop predictive models for leaf turgor pressure by employing meteorological parameters and plant physiological indicators (Bader et al., 2014; Barriga et al., 2022; Camoglu et al., 2021; Fernandes et al., 2017). This research aims to facilitate the accurate estimation of plant water conditions. Nevertheless, modeling should account for soil moisture fluctuation as it is the relatively controllable variable in commercial agriculture (Kabala et al., 2025; Velazquez-Chavez et al., 2024). Thus, it is essential to investigate the quantitative correlations between weather, soil moisture, and leaf turgor pressure. This investigation will provide the foundation for constructing predictive models of leaf turgor pressure for greenhouse tomato and celery.

Machine learning techniques have demonstrated distinct advantages in the study of complex environment–physiology coupling by virtue of their powerful nonlinear modeling capability and efficient computational performance. In particular, techniques such as Support Vector Machine (SVM), Extreme Gradient Boosting (XGBoost), and Random Forest (RF) are often favored for their high prediction accuracy. Accordingly, these techniques are frequently employed in predicting plant water status through the analysis of diverse input parameters, including remote sensing spectral indices, soil physicochemical characteristics, and climatic elements (Amir et al., 2021; Gao et al., 2023; Guimarães et al., 2024; Shi et al., 2022). Collectively, these studies underscore the effectiveness of SVM, RF, and XGBoost in assessing the environmental response of plant physiology and developing irrigation schedules for plants.

Addressing the aforementioned challenges and leveraging the strengths of machine learning, the present study investigated greenhouse tomato and celery under various drip irrigation treatments by monitoring leaf turgor pressure, meteorological factors, and soil moisture content. Subsequently, machine learning techniques were employed to develop models for leaf turgor pressure prediction, aiming to provide useful insights for drip irrigation scheduling in greenhouse vegetables. The specific objectives of this study are as follows: (1) to examine the transient and interday characteristics of tomato and celery leaf turgor pressure under various moisture conditions, (2) to explore the response relationship between leaf turgor pressure and environmental factors, and (3) to construct a long-time series prediction model for greenhouse tomato and celery leaf turgor pressure in drip irrigation using SVM, XGBoost, and RF.

## 2 Materials and methods

### 2.1 Experimental site and experimental design

The experimental site is situated in Liujiabao Village, Taiyuan City, Shanxi Province (112°29′E 37°39′N, altitude 766 m), which has a mild temperate continental climate. The region experiences a summer concentration of rainfall, with a multiyear average precipitation of 495 mm. It has an annual frost-free period of 202 d, an average annual temperature of 9.6°C, and average annual sunshine hours of 2675.8 h. Drip irrigation experiments were conducted in a naturally ventilated solar greenhouse during summer and winter. The greenhouse had an east–west orientation, with a transverse and longitudinal diameter of 60 m × 11 m. The soil layer from 0–60 cm was silty loam, with an average bulk density of 1.45 g·cm^-3^. The soil field capacity (θ_f_) was 32% for tomato experimental plot and 38% for celery experimental plot.

**Figure 1:**
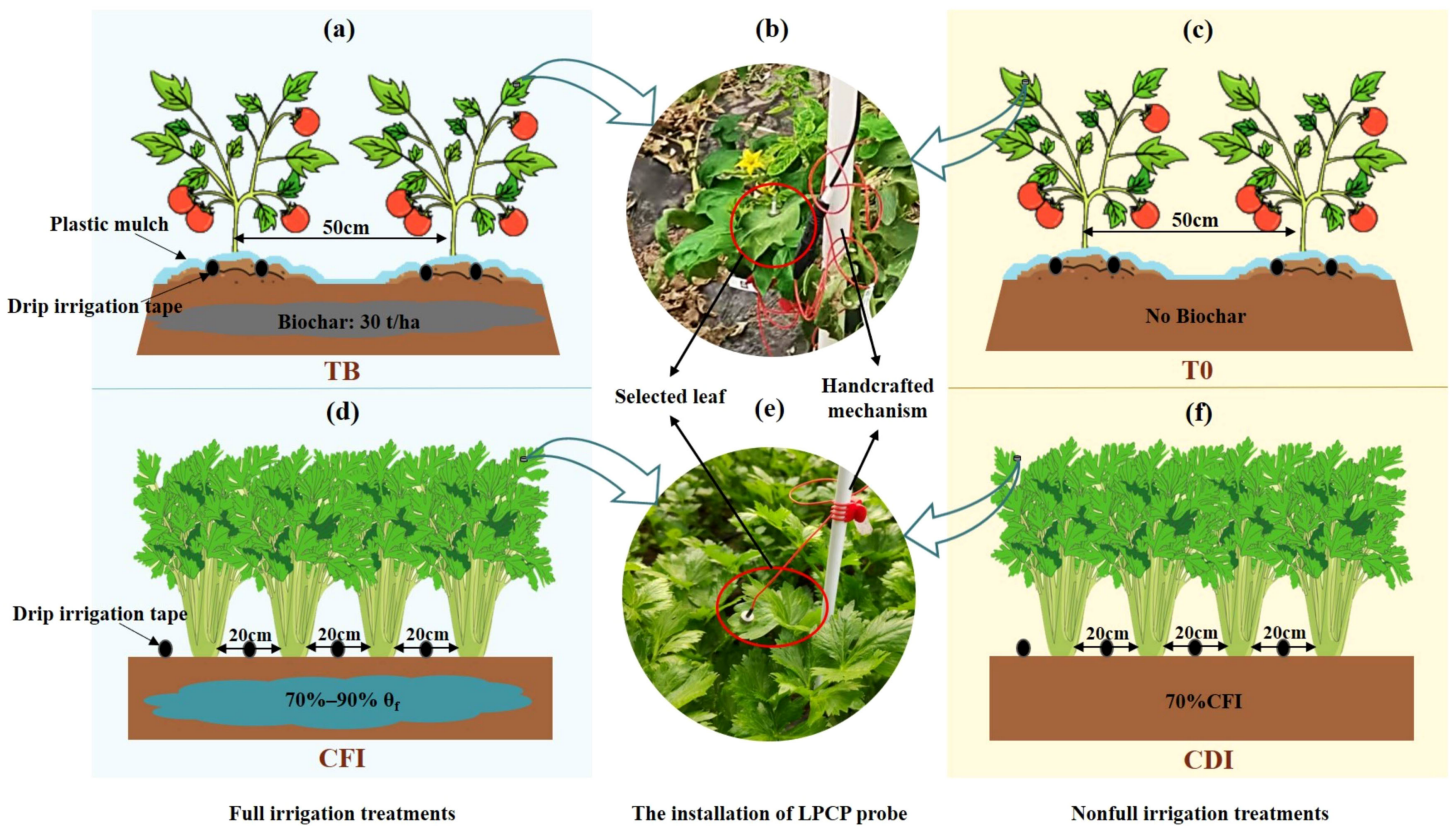
Field layout diagram **(a, c, d, f)** and LPCP probe installation diagram **(b, e)** of tomato and celery experiment.

The summer crop selected for the experiment was the ‘Shouyan PT 326’ tomato. This experiment used a modification in biochar addition as the principal approach to adjust tomato irrigation treatments, rather than changing irrigation time or volume. The strategy was based on the previous study, which found that incorporating biochar into soil improves its water retention capacity (Wei et al., 2023). Specifically, the full irrigation treatment (TB, **Figure 1a**) received an initial application of corn stalk biochar (produced by high-temperature pyrolysis, 400–500°C) incorporated into the top 0–30 cm soil layer in 2021, alongside 20,000 kg·ha^-^¹ of cow manure, with subsequent annual applications of the same quantity of cow manure. Meanwhile, the non-full irrigation treatment (T0, **Figure 1c**) had no biochar addition (**Supplementary Table S1** provides the fundamental physicochemical properties of the 0–30 cm soil layer for TB and T0 treatments). The planting periods for tomato were May 17, 2021, to Sept. 19, 2021, and May 25, 2022, to Sept. 30, 2022. And tomatoes were grown in rows 60 cm × 50 cm apart, with two drip irrigation tapes laid out for each row.

The winter crop used for the experiment was ‘France Queen’ celery. As opposed to tomato, the celery might be grown with full or non-full irrigation by adjusting the amount of water used. The irrigation range for the full irrigation celery treatment was defined as 70%–90% of θ_f_ (CFI, **Figure 1d**), and ‘I’ stood for a single irrigation volume. Conversely, 70%I was chosen as the irrigation volume for the non-full irrigation treatment (CDI, **Figure 1f**). The planting periods for celery were Nov. 1, 2021, to Jan. 19, 2022, and Nov. 15, 2022, to Feb. 11, 2023. The celery was grown in rows 20 cm × 20 cm apart, with one drip irrigation tape laid out for each row. Before celery transplanting, each treatment was supplied with 888 kg·hm^-^² of NPK compound fertilizer as a basal application, uniformly incorporated into the plough layer. Other management practices, such as pesticide application and weed control, were kept consistent across all treatments throughout the entire growing season.

Drip irrigation tapes were arranged in a patchwork configuration, with a working pressure ranging from 1.0 to 2.0 MPa, a rated flow rate of 1.38 L·h^-1^, and a drip head spacing of 30 cm. To increase the survival rate of seedlings, tomato was irrigated with 25-mm planting water, and celery was irrigated with 50-mm planting water and 36-mm seedling water. Subsequently, different experimental treatments were conducted (**Table 1**). During the test, pest management and fertilization were performed according to local customs.

**Table 1:** Drip irrigation details of greenhouse tomato and celery. The irrigation schedule for tomatoes includes one planting irrigation. The celery irrigation schedule consists of one planting irrigation and one slow seedling irrigation.

Treatment		2021		2022	
		Irrigation amount/mm	Schedule	Irrigation amount/mm	Schedule
TB	Biochar: 30 t/ha	226.8	6	264.6	7
T0	No biochar	226.8	6	264.6	7
		2021-2022		2022-2023	
CFI	70%–90% θf	180	7	144.6	4
CDI	70% CFI	153	7	121.3	4

### 2.2 Determination of leaf turgor pressure

In this experiment, the leaf patch clamp pressure (LPCP, YARA-ZIM Plant Technology GmbH, Germany) probes were employed to determine the output parameter (P_p_). The magnitude of P_p_ is inversely proportional to the change in leaf turgor pressure; therefore, a high value implies low leaf turgor pressure. Three healthy plants exhibiting uniform growth characteristics were randomly selected from each of the two experimental treatments. For each selected plant, a single, mature, and fully expanded leaf, located in the upper canopy (within the uppermost 30% of the plant’s height), was chosen for probe installation. This exposed leaf was confirmed to be east-facing, and showed no signs of pest infestation, disease, or physical damage. To maintain probe stability post-installation, leaves were supported by handcrafted mechanisms designed to provide gentle, passive support without compromising leaf integrity or physiology (**Figures 1b, e**). Data were logged every 5 minutes throughout the growth period. The detail of growth stage division for monitoring leaf turgor pressure in tomato and celery were presented in **Supplementary Table S2**.

In this study, the P_p_ diurnal variation curve is divided into two states: State I and State II. State I (unimodal pattern): the P_p_ diurnal variation curve exhibits a single principal maximum, typically occurring around midday, with minimum values observed at night. The diurnal cycle in State I can be characterized by four sequential stages: valley fluctuation stage, rapid rising stage, peak fluctuation stage, and decay stage (Xu et al., 2024). State II (troughed pattern): the P_p_ diurnal variation curve exhibits a pronounced daytime minimum, which fundamentally defines the observed ‘N’, ‘M’, or ‘V’ pattern. This pattern is further characterized by the occurrence of multiple periods of suppressed values and potentially several distinct phases of elevated values. The curve exhibits a variable number of extrema (both maxima and minima) throughout the day. The determination of P_p_ states was achieved through a meticulous two-step process. Preliminarily, the identification was conducted by calculating the mean P_p_ rise rate from 5:00 to 10:00 and the mean P_p_ fall rate from 15:00 to 20:00. If both rates were negative, it was classified as a ‘V’ pattern within State II; if any rate was negative, it was classified as an ‘N’ pattern within State II; and if both rates were positive, the preliminary classification of P_p_ was designated as State I. Subsequently, manual screening via graphing was conducted to identify and correct any omissions. The culmination of this study revealed that P_p_ demonstrated State II for a duration of 97 days. Among these, the proportions of ‘N’, ‘M’, and ‘V’ shaped curves accounted for 36.08%, 30.93%, and 35.05%, respectively.

### 2.3 Determination of environmental factors

Meteorological data were collected from a fully automated weather station inside the solar greenhouse. Monitoring indicators included net solar radiation (Rs, W·m^-2^; measured by Li-200R pyranometer, LI-COR, USA), atmospheric temperature (T, °C), relative humidity (RH, %; measured by ATMOS-14 sensor, METER, USA), and wind speed (WS, m·s^-1^; measured by ATMOS-22 sensor, METER, USA). All variables were collected and saved by a data logger (CR1000, Campbell, USA) at 5-minute intervals. The saturated water vapor pressure deficit (VPD, kPa) was calculated using the Equation 1 (Campbell and Norman, 1999).

(1) $$VPD=0.611\times \exp\left(\frac{17.502\times T}{T+240.97}\right)\times (1-RH)$$

Soil water content (SWC, %) was measured at soil depth of 0–60 cm with a 10 cm interval using a time-domain reflectometry (TRIME-PICO IPH TDR, IMKO, Germany). Access tubes were installed in each experimental plot before the experiment commenced. Measurements were taken approximately every 7 days, with additional measurements conducted immediately before and after irrigation events. During the experiment, the volumetric water content measured by the time-domain reflectometry was periodically calibrated using the oven-drying method.

### 2.4 Statistical analysis

SPSS 24 and OriginPro 2021 software were used for data processing and visualization in this investigation. The independent-samples t-test and one-way ANOVA were used to assess the significance of differences across treatments. The Duncan technique was used for multiple comparisons (*P* < 0.05).

Universal transient leaf turgor pressure prediction models for tomato and celery were developed using SVM, XGBoost, and RF techniques. These algorithms were chosen for their effectiveness in complex regression tasks, particularly with potentially limited sample sizes (Zenone et al., 2022; Lu et al., 2023; Garofalo et al., 2024). For model development, the intermittent soil water content data were linearly interpolated to obtain daily values. It was hypothesized that the daily values of soil water content would remain constant for 24 hours (Cheng et al., 2017). Subsequently, P_p_ and environmental parameter data were preprocessed to generate a continuous dataset with a 5-minute time scale. Model building and parameter optimization were performed using the scikit-learn and XGBoost libraries of Python 3.7 (Python Software Foundation, Wilmington, Delaware, USA). The modeling used 80% of the input data for training and the remaining 20% for testing. Furthermore, the two P_p_ states’ data could either be integrated into a single state (whole state P_p_) or separated (substate P_p_) during modeling, allowing for flexible analysis. The performance of the models was evaluated using several metrics, including the coefficient of determination (R2, Equation 2), mean square error (MSE, Equation 3), root mean square error (RMSE, Equation 4), and mean absolute error (MAE, Equation 5).

(2) $$R^{2}=1-\frac{\sum_{i=1}^{n}{\left(Y_{i}-y_{i}\right)}^{2}}{\sum_{i=1}^{n}{\left(Y_{i}-\bar{Y}\right)}^{2}}$$

(3) $$MSE=\frac{\sum_{i=1}^{n}{\left(Y_{i}-y_{i}\right)}^{2}}{n}$$

(4) $$RMSE=\sqrt{\frac{1}{n}\sum_{i=1}^{n}{\left(Y_{i}-y_{i}\right)}^{2}}$$

(5) $$MAE=\frac{\sum_{i=1}^{n}\left|Y_{i}-y_{i}\right|}{n}$$

where n is the number of samples, $y_{i}$ and $Y_{i}$ are the predicted value and the measured value, respectively; and $\bar{Y}$ is the average measured value.

## 3 Results

### 3.1 Characteristics of environmental changes during the test period

**Figure 2:**
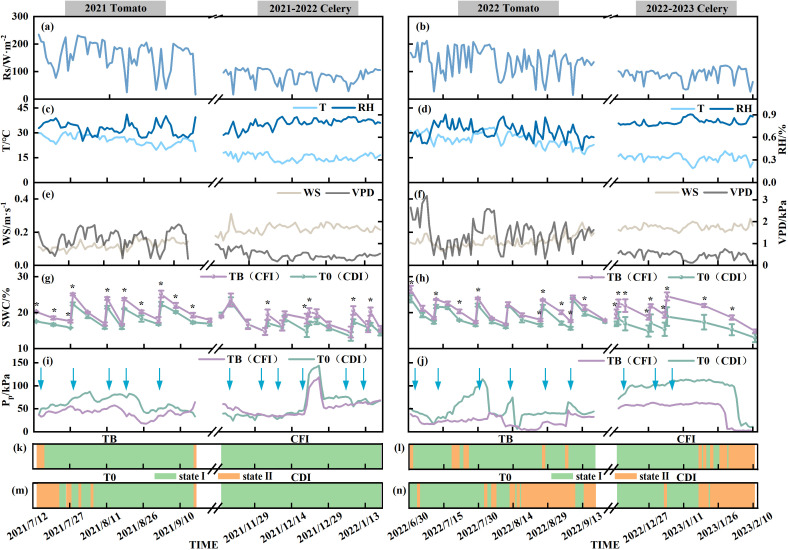
Interdaily variations of environmental factors and P_p_ during the 2021–2023 test periods, with the 2021–2022 test period on the left and the 2022–2023 test period on the right. The environmental factors include Rs **(a, b)**, T, RH **(c, d)**, WS, VPD **(e, f)**, and SWC **(g, h)**. The variations in P_p_ include daily means of P_p_ for tomato and celery (i and j), P_p_ states for TB and CFI treatments **(k, l)**, and Pp states for T0 and CDI treatments (m and n). The blue arrow indicates irrigation **(I, j)**. * indicates *P<* 0.05.

The tomato experiment was conducted in summer and autumn, whereas the celery examination was conducted in winter and spring (**Figure 2**). In comparison to winter and spring, the average Rs, T, and VPD in the greenhouse were higher in summer and autumn, with more variability (CV = 11.01%–45.65% for tomato, CV = 5.07%–40.88% for celery). Over the study period, the average RH and WS during the celery trial were 9.75%–83.56% higher than those during the tomato trial, whereas the average Rs, T, and VPD were 34.77%–67.39% lower (*P* < 0.01). Interannual analysis revealed that for tomato, the daily mean WS and VPD in 2022 were significantly lower by 16.76% and 17.66%, respectively, compared to 2021. The daily mean Rs and RH in 2022 were significantly higher by 5.40% and 16.08%, respectively (*P* < 0.05). No notable interannual variations in weather were observed during the celery trial. Meanwhile, **Figures 2g, h** show that there were nonsignificant interannual variations in SWC between the respective treatments for each vegetable. In addition, comparing the treatments showed that TB’s SWC markedly exceeded that of T0 (*P* < 0.05). Similarly, the SWC under CFI was considerably higher than under CDI, with an average increase of 24.37% during the 2022–2023 period.

### 3.2 Characterization of leaf turgor pressure changes in drip-irrigated tomato and celery

**Figure 3:**
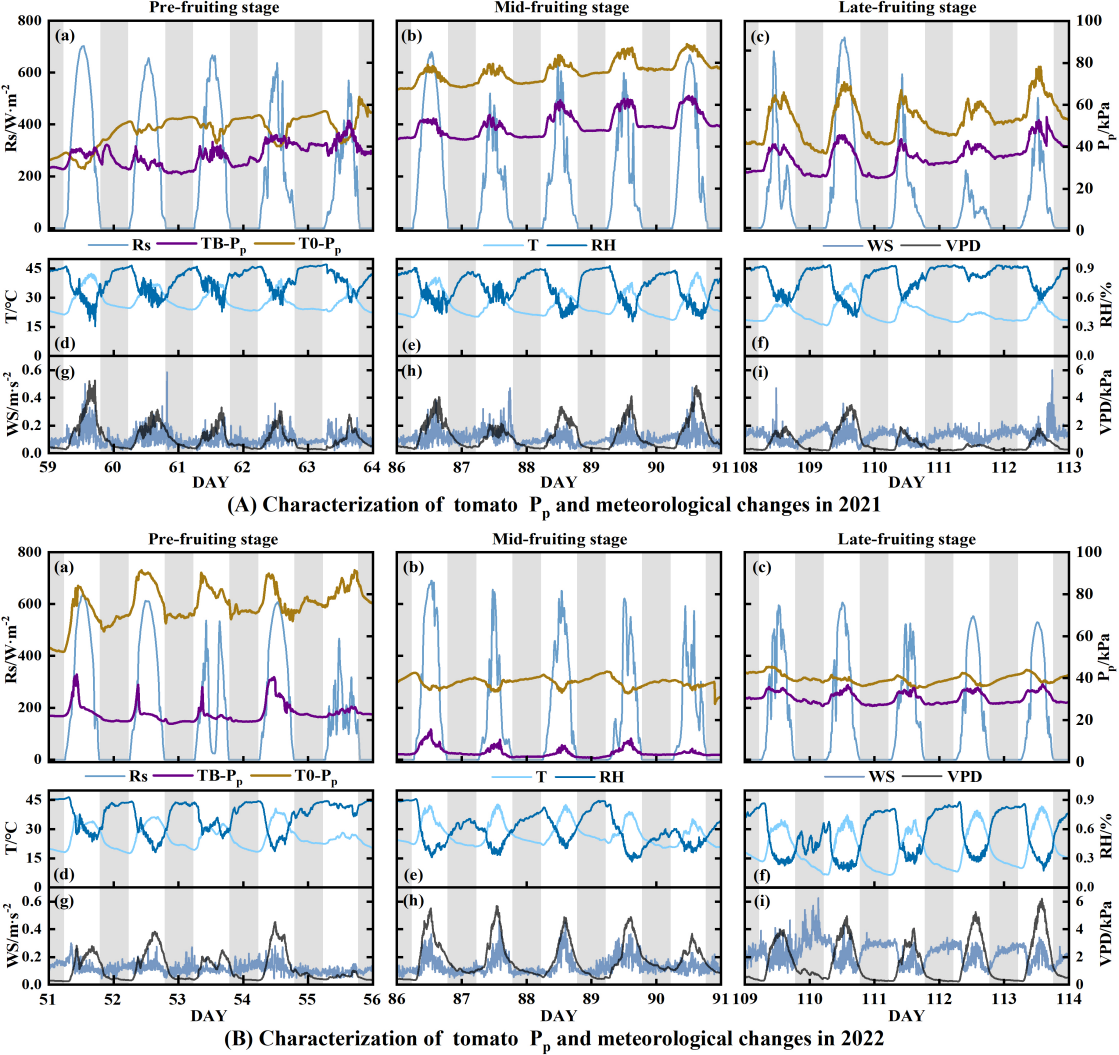
Daily changes in P_p_ and meteorological factors (Rs, T, RH, WS, and VPD) for tomato during the pre-fruiting (a, d, and g), mid-fruiting (b, e, and h), and late-fruiting (c, f, and i) stages in 2021 and 2022. **(A)** 2021; **(B)** 2022. The word ‘DAY’ refers to the number of days following planting.

**Figure 4:**
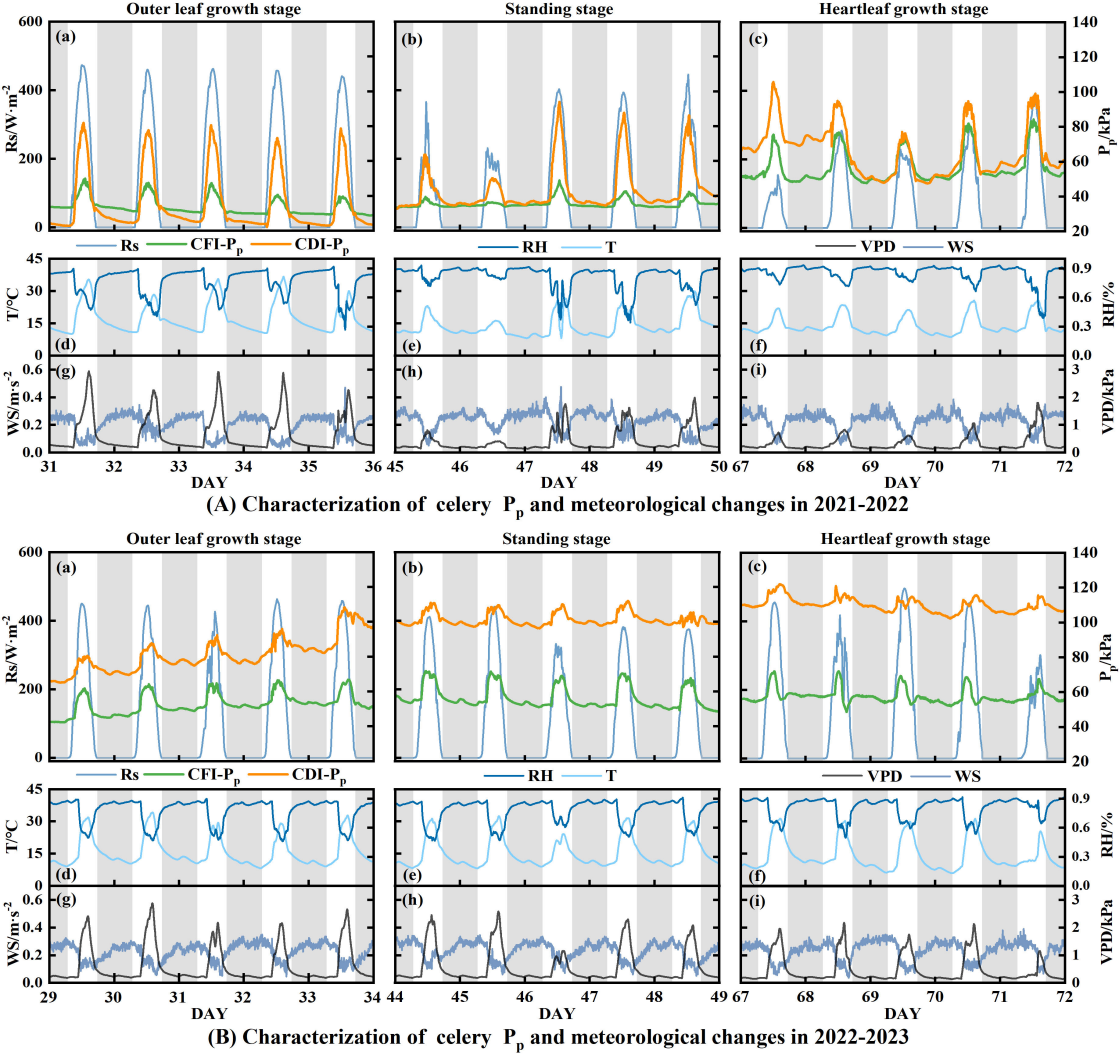
Daily changes in P_p_ and meteorological factors (Rs, T, RH, WS, and VPD) for celery during the outer leaf growth (a, d, g), standing (b, e, h), and heartleaf growth (c, f, i) stages from 2021 to 2023. **(A)** 2021–2022; **(B)** 2022–2023. The word ‘DAY’ refers to the number of days following planting.

The daily P_p_ variation curves for greenhouse celery leaves and tomato fruits during their growth phases demonstrated two prevalent modes: State I, a unimodal diurnal variation, and State II, troughed diurnal variation (**Figures 2**–**4**). Within State I, the P_p_ showed a diurnal pattern, peaking at midday (P_p,max_) and falling at night (P_p,min_). However, it exhibited P_p,max_ at night and P_p,min_ during the day in State II. State I appeared across all stages of fruit growth under different tomato treatments, but State II appeared mainly in the pre-fruiting stage of 2021 and mid- and late-fruiting stages of 2022. The percentages of State I for the TB treatments were 93.85% in 2021 and 89.74% in 2022. By contrast, T0 treatment had a lower State I occupancy of 15.39% (2021) and 30.05% (2022) than those of TB. Moreover, across both years, the P_p,max_ of T0 was 48.85%–109.14% higher than that of TB, whereas the P_p,min_ of T0 was 75.84%–138.39% higher than that of TB. Concurrently, the P_p_ curves exhibited an upward trajectory, concomitant with fruit expansion during the irrigation period. From DAY86 to DAY91 (2021), P_p,max_ increased by 9.78 kPa (T0) and 10.76 kPa (TB), while P_p,min_ increased by 9.39 kPa (T0) and 5.28 kPa (TB) (**Figure 3**).

The proportion of P_p_ states remained constant during the experimental period across different celery treatments. During 2021–2022, both CFI and CDI treatments remained in State I. In 2022–2023, two states were observed, with State II accounting for 30% of the observation time for CF and 41.67% for CD. State II primarily appeared in the late heartleaf growth stage. However, the mean daily P_p_ of CDI in both experimental periods exhibited an increasing tendency as the celery leaves grew and developed (**Figures 2i, j**). For the CDI treatment, daily P_p_ variations were more pronounced than in CFI. Specifically, compared to the mean daily P_p,max_ and P_p,min_ of the CFI treatment, the mean daily values for CDI when in State I were 34.98% higher for P_p,max_ in 2021–2022 and 66.74% higher in 2022–2023, and 3.44% higher for P_p,min_ in 2021–2022 and 94.02% higher in 2022–2023. The P_p_ curves of the CFI treatment for the 2021–2022 period exhibited a gradual increase as the growth period progressed (**Figure 4**). By contrast, the P_p_ variation remained relatively stable across the three growth periods from 2022 to 2023, except for a decrease in mean daily P_p_ that occurred during the late heartleaf growth stage (**Figure 2j**).

### 3.3 Influencing factors of leaf turgor pressure in drip-irrigated tomato and celery

**Figure 5:**
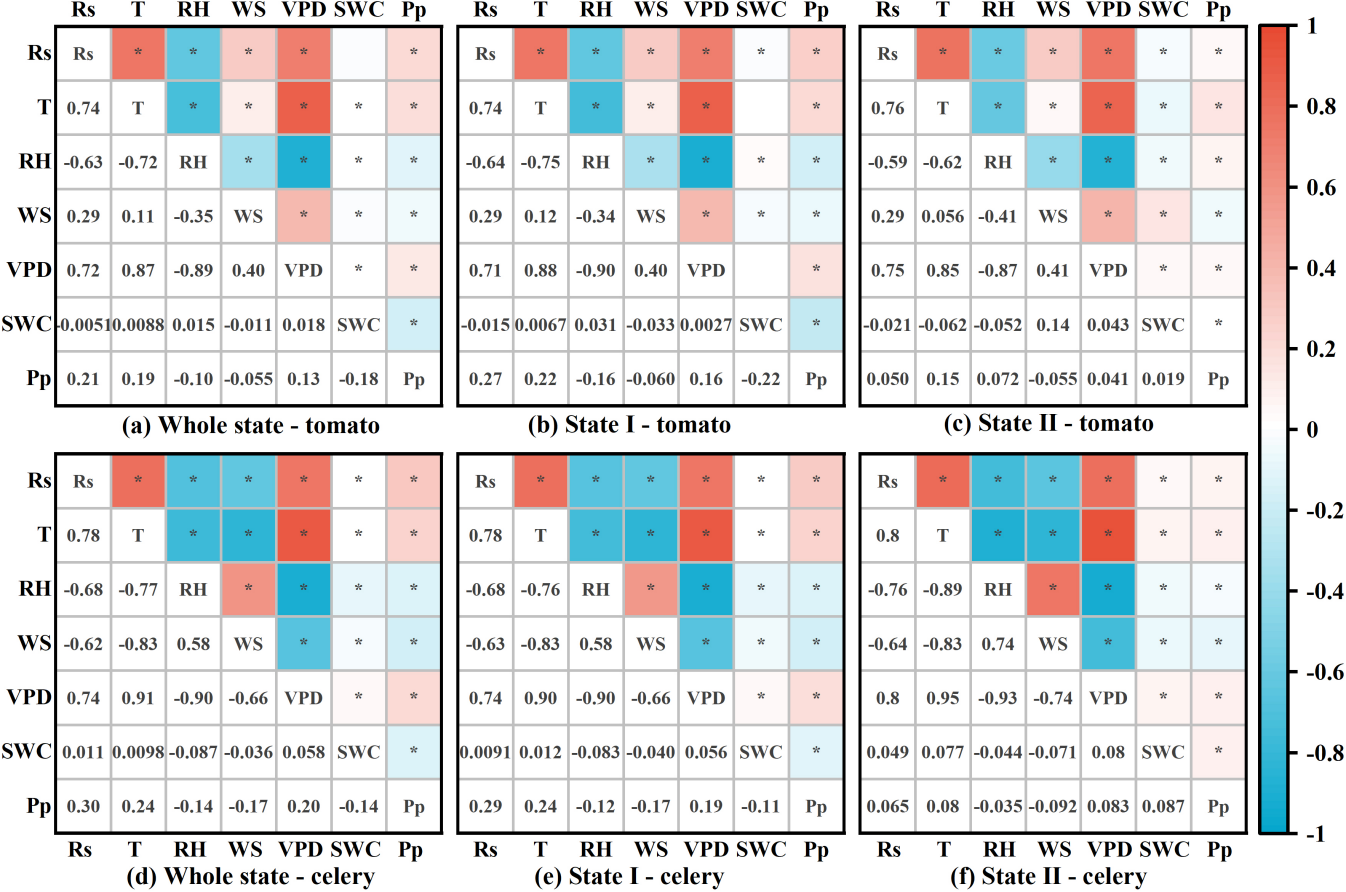
Heatmaps of the correlation between instantaneous-scale P_p_ and environmental factors throughout the growth cycles of tomato and celery. Figures **(a–c)** show the correlations for tomato in the whole state, State I, and State II, respectively. Figures **(d–f)** show the correlations for celery in the whole state, State I, and State II, respectively. * indicates *P* < 0.05.

The daily trend indicated that the higher the daily maximum of Rs, T, and VPD, the greater the associated P_p,max_ (**Figures 3**–**5**). The P_p_ of full irrigation treatments (TB, CFI) was significantly lower than that of non-full irrigation treatments (T0, CDI). For instance, the daily average P_p_ for TB was 26.21 kPa in 2021 and 22.81 kPa in 2022, both of which were lower than those observed for T0. There was a definite trend of decreasing P_p_ in the TB, T0, and CDI after irrigation, followed by a gradual rise throughout the irrigation cycle (**Figure 2**). Therefore, in the whole state and State I, P_p_ was positively correlated with Rs, T, and VPD but negatively correlated with RH, WS, and SWC (*P* < 0.05). However, it was observed that while daily averages of environmental factors showed strong correlations with instantaneous P_p_, this relationship attenuated when considering the entire growth period. This suggests that a straightforward linear connection could not adequately describe the effect of environmental changes on P_p_.

**Figure 6:**
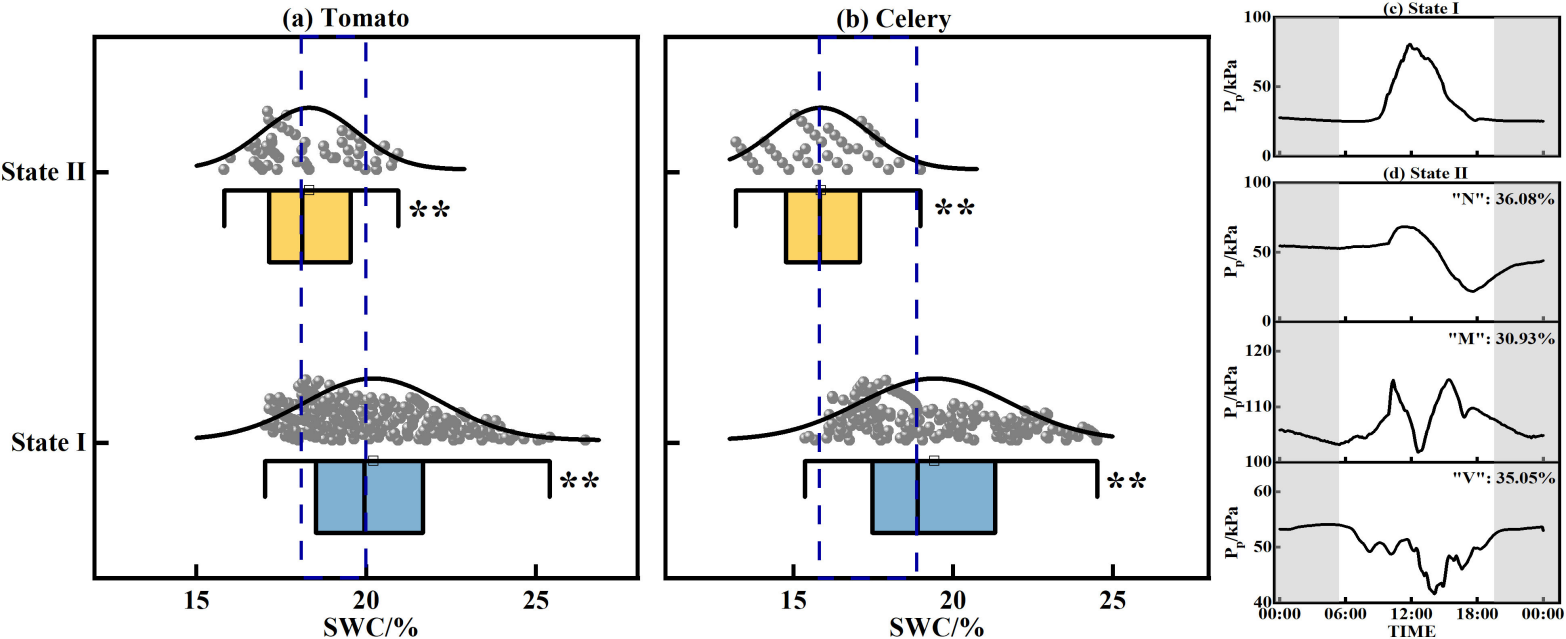
Changes in SWC corresponding to the different states of greenhouse tomato and celery in drip irrigation; **(a)** tomato; **(b)** celery; **(c)** diurnal variation of P_p_ in state I; **(d)** diurnal variations of P_p_ in state II. The dashed lines denote the boundaries delineating states I and II of SWC. ** indicates *P* < 0.01.

The daily pattern of P_p_ change serves as an indicator of soil moisture variability. **Figure 6** depicts the SWC variations over different states during the experiment. Specifically, the average SWC in State II was 18.32% for greenhouse tomato and 15.86% for celery, which were lower than those in State I. Additionally, P_p_ state and SWC were combined to split the threshold. For greenhouse tomato, State I of P_p_ was consistently observed when SWC exceeded 20%, while State II was characteristic of SWC below 18%. For celery, State I was observed with SWC > 19%, and State II with SWC< 16%. However, between 18% and 20% SWC for tomato, and between 16% and 19% SWC for celery, the classification of P_p_ into State I or State II was less definitive, with instances of both states being observed.

### 3.4 Predictive modeling of tomato and celery leaf turgor pressure under drip irrigation

According to the above analysis results, four combinations of inputs and outputs were established to predict leaf turgor pressure based on the characteristics and influencing factors of P_p_ in different states of greenhouse tomato and celery (**Table 2**): Combination 1 (whole state P_p_ prediction based on meteorological factors), Combination 2 (substate P_p_ prediction based on meteorological factors), Combination 3 (whole state P_p_ prediction based on meteorological factors and SWC), and Combination 4 (substate P_p_ prediction based on meteorological factors and SWC).

**Table 2:** The parameter combinations of predictive models.

Combination	Description	
	inputs	outputs
1	Rs, T, RH, WS, VPD	whole state P_p_
2	Rs, T, RH, WS, VPD	substate P_p_
3	Rs, T, RH, WS, VPD, SWC	whole state P_p_
4	Rs, T, RH, WS, VPD, SWC	substate P_p_

**Figure 7:**
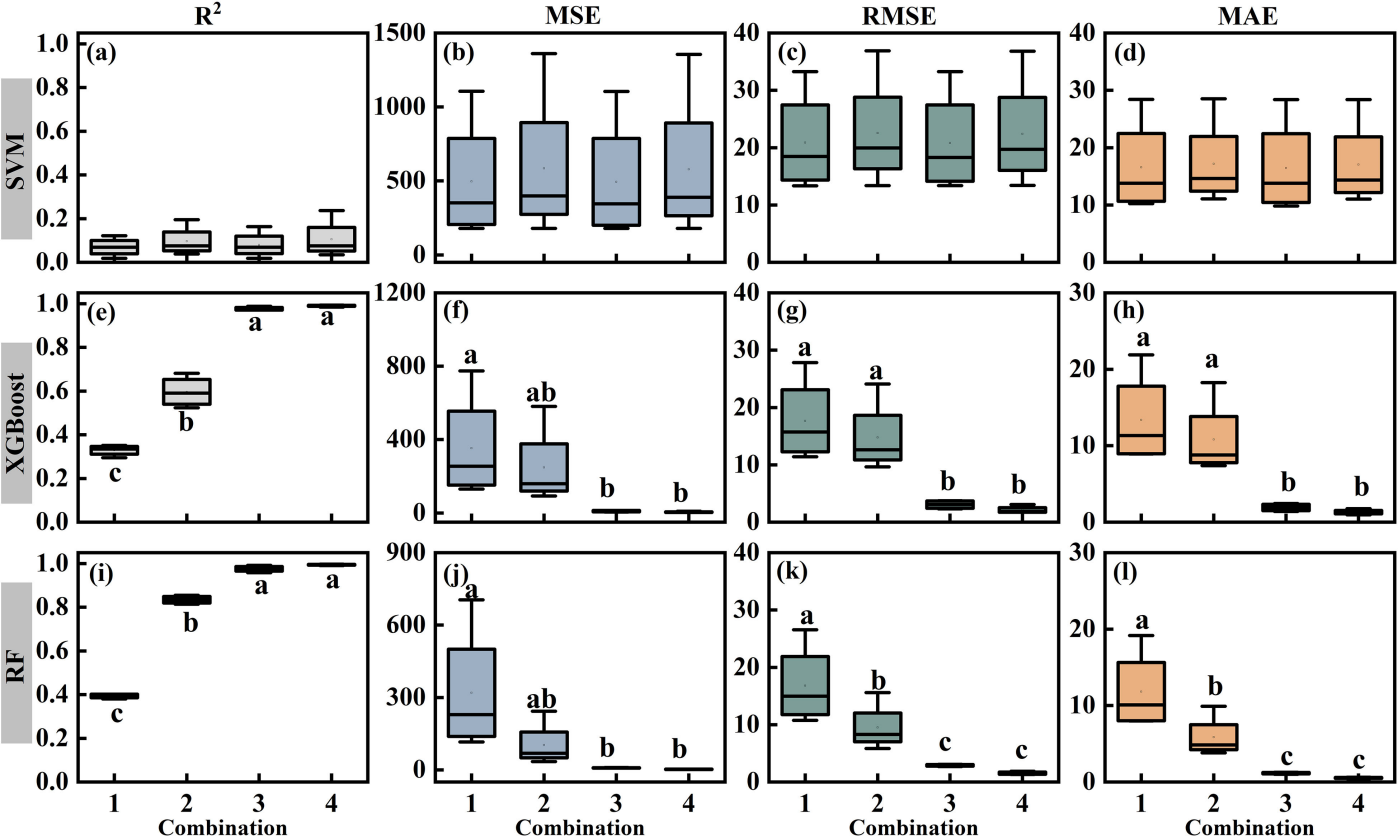
Alterations in the evaluation indices (R^2^, MSE, RMSE, and MAE) of all irrigated tomato and celery treatments under three techniques (SVM **(a–d)**, XGBoost **(e–h)**, and RF **(i–l)**). Lowercase letters indicate significant differences between combinations (*P* < 0.05).

**Figure 8:**
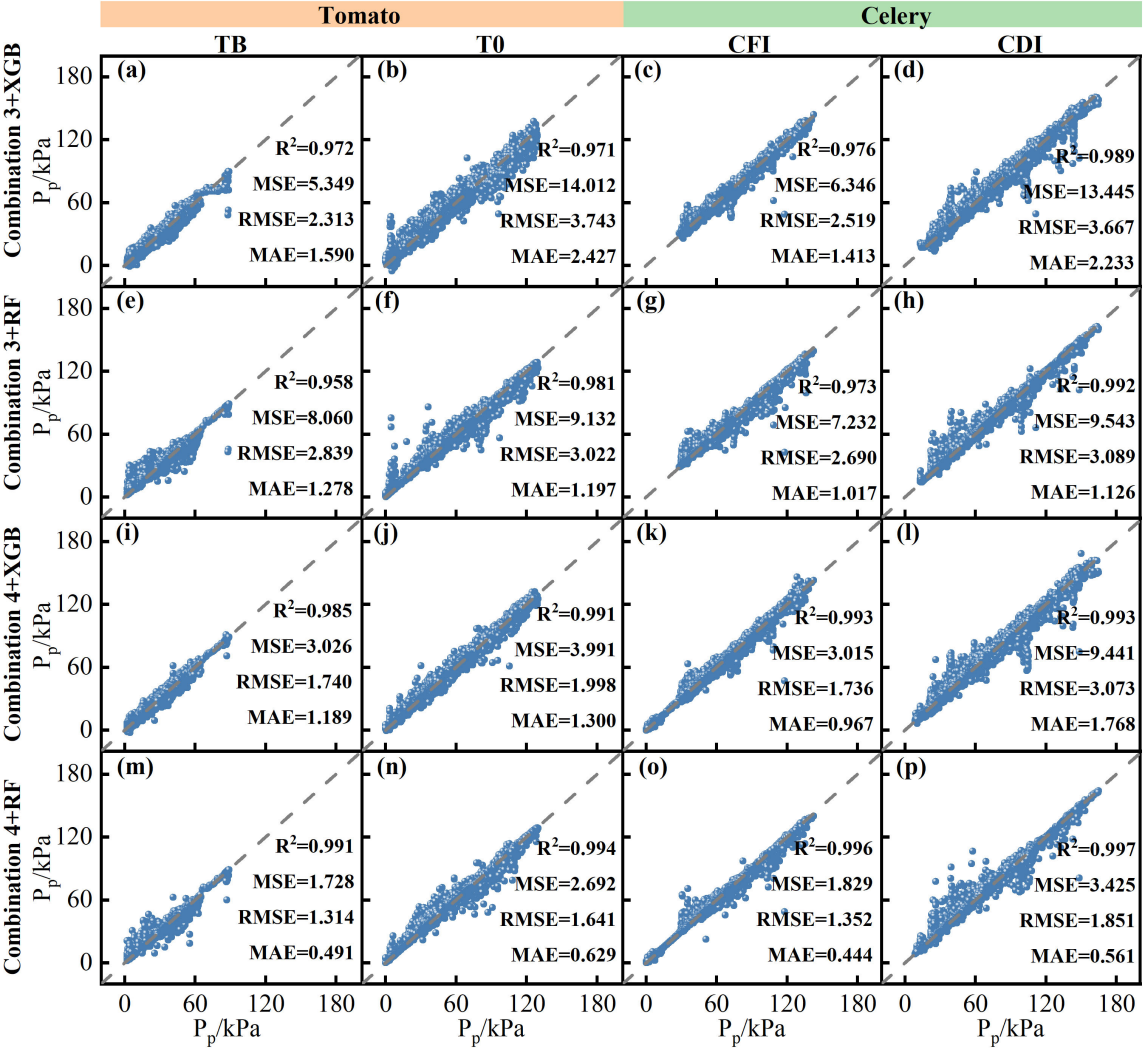
Comparison of simulated versus measured values for various treatments (TB, T0, CFI, and CDI) in Combinations 3 and 4 using the XGBoost and RF techniques [**(a–d)**. Combination 3 and XGBoost; **(e–h)**. Combination 3 and RF; **(i–l)**. Combination 4 and XGBoost; **(m–p)**. Combination 4 and RF].

**Figure 7** presents the assessment and prediction outcomes of all irrigated tomato and celery treatments for various combinations. The performance of the SVM had low R² values that showed negligible improvement across different input combinations. In contrast, both XGBoost and RF exhibited significantly improved performance when predictions were based on substate P_p_ data and included SWC as an input parameter. Specifically, for these models, R² values increased significantly (*P* < 0.05), while the error metrics (MSE, RMSE, and MAE) decreased. When the prediction outcomes of various treatments were further compared, all P_p_ prediction models developed using Combinations 3 and 4 with XGBoost and RF had R^2^ values of > 0.95 (**Figure 8**). Among these, the RF model using Combination 4 was identified as the optimal model for predicting leaf turgor pressure in both tomato and celery. The average value of R^2^, MSE, RMSE, and MAE for the best model was 0.995, 2.418, 1.540, and 0.531, respectively. Analysis of feature importance within this optimal model (**Supplementary Figure S1**) indicated that SWC was the most influential parameter in both State I and State II.

## 4 Discussion

### 4.1 Characterizing changes and influencing factors of leaf turgor pressure in drip-irrigated tomato and celery

In this study, the LPCP probe was employed to elucidate the characteristics of leaf turgor pressure changes at different growth stages of greenhouse tomato and celery in drip irrigation. During the entire growth period, the daily pattern of P_p_ exhibited two distinct states: a unimodal pattern (I) and a troughed pattern (II). In State I, P_p_ reached a maximal value observed during daylight hours and a minimal value during the night (**Figure 6c**). In this state, meteorological parameters were the primary determinants of the daily leaf turgor pressure pattern. The main causes of transpirational water loss, which reduce leaf turgor pressure (P_p_ rises), were determined to be Rs, T, and VPD. By contrast, RH and WS have been shown to inhibit stomatal opening, increasing leaf turgor pressure (P_p_ drops) (Hsie et al., 2015). Rs was found to exert the most significant influence on leaf turgor pressure in greenhouse tomato and celery, which resulted in lower mean daily P_p_ values on overcast or wet days than on sunny days. Moreover, SWC influenced the interday fluctuation of leaf turgor pressure. Specifically, in State I, non-full irrigation treatments (T0, CDI) resulted in higher average P_p,max_ and P_p,min_ compared to the full irrigation treatment (TB, CFI). This phenomenon could be mainly attributed to the decline in soil water supply capacity. Consequently, the plant’s root water absorption was unable to counteract transpiration water loss, leading to a decrease in leaf turgor pressure (Holz et al., 2024). The decline in leaf turgor pressure coincided with the steady depletion of soil moisture. Some studies have also shown that the time for P_p_ to decrease from P_p,max_ to P_p,min_ in State I increased with the decrease in SWC; however, the leaf turgor pressure still demonstrated the ability to recover (Zimmermann et al., 2013). This resilience indicates that plants in State I are tolerant to drought stress.

State II exhibits a complex diurnal curve that significantly differs from State I. The curve of State II, characterized by bimodal/multimodal maxima or a distinct diurnal minimum, gives rise to ‘N’, ‘M’, or ‘V’-shaped patterns (**Figure 6d**). Relevant researchers had posited that the emergence of the anomalous curve is associated with the increased gas content in the leaf (Ehrenberger et al., 2012). Under conditions of escalating soil moisture deficit, plants experience reduced leaf turgor pressure and/or elevated abscisic acid (ABA) accumulation, which is a key mediator of stomatal closure (McAdam and Brodribb, 2016; Mo et al., 2025). This closure limits CO_2_ diffusive flux. Concurrently, drought stress directly hinders CO_2_ uptake through mechanisms such as leaf shrinkage, changes in bicarbonate homeostasis, and impaired aquaporin function (Hao et al., 2023), all leading to increased intercellular CO_2_ concentrations. Moreover, meteorological drought independently provokes stomatal closure, further elevating internal CO_2_ levels (Williams et al., 2022). Therefore, the LPCP probe signals during State II primarily reflect bulk changes in internal CO_2_ volume rather than fine-scale variations in leaf turgor. This finding suggests that State II can serve as a reliable marker for meteorological drought conditions and soil water stress. In this experiment, State II under full irrigation was most noticeable during the pre-fruiting stage (2021), mid- and late-fruiting stages (2022), and late heartleaf growth stage (2022–2023). The daily average Rs in State II was significantly higher than that in State I (*P* < 0.05), despite lower SWC. This phenomenon suggests that meteorological drought stress causes P_p_ reversal even under full irrigation. Furthermore, within the controlled greenhouse environment, non-full irrigation treatments led to concurrent reductions in soil moisture and atmospheric humidity, causing dual stresses of soil and meteorological drought and subsequently increasing the frequency of State II (**Figure 2**). However, Cabrita (2022) found that *Hippeastrum* P_p_ remained in State II under conditions of no water stress. This phenomenon could be attributed to the presence of aerenchyma, which comprises a substantial number of cavities (63% by volume) within the leaf pulp of Hippeastrum. When stomata open, humid air enters the leaf, elevating the air pressure within the ventilation tissue above ambient levels. Consequently, LPCP probes predominantly detect the pressure changes within the aerenchyma. In addition, the leaf turgor pressure irrigation response exhibited greater strength in the nonfull irrigation treatment, as evidenced by the substantial reduction in P_p_ after irrigation (**Figures 2i,j**). The response could be attributed to the diminished plant water potential under non-full irrigation. This condition created a strong water potential gradient that drove an increased rate of root water uptake upon reirrigation, leading to a swift recovery of leaf turgor pressure (Hao et al., 2019). Collectively, these findings indicate that the P_p_ curve effectively captures distinct water status characteristics of greenhouse tomato and celery, indicating its utility for diagnosing crops water status.

However, the characterization of the P_p_ state may slightly differ among plant species. Fernández et al. (2011) and Ehrenberger et al. (2012) found that olive trees exhibit a semi-inverted daily variation in P_p_, intermediate between State I and State II. This phenomenon could be attributed to the enhanced resilience of trees, which stems from thicker cell walls in their xylem conduit tubes. This structural adaptation mitigates the risk of embolization and ensures a reliable water supply to the leaves (Sperry and Love, 2015). Furthermore, trees employ an ‘isohydric’ water use strategy, which precisely regulates stomatal dynamics and mitigates transpirational water loss (McDowell et al., 2008). Additional traits, such as a thick cuticle, low stomatal density, and a waxy cover of tree leaves, enhance water retention and reduce water loss (Iqbal et al., 2017). Collectively, these factors increase the semi-inversion frequency by causing the P_p_ of trees to shift from unimodal to inverted conditions over extended periods during water stress. By contrast, tomato and celery exhibit increased water sensitivity, characterized by a rapid increase in stomatal closure and intercellular CO_2_ concentration during periods of augmented water stress (Li et al., 2022; Xu et al., 2022). This increased sensitivity results in a direct shift of the P_p_ curves from State I to State II, a transition that can be used as a reference signal for irrigation. In related research, P_p_ status was analyzed alongside midday stem water potential (Ψ_stem_) to establish irrigation thresholds. For young and mature olive trees, these reference thresholds were determined to be Ψ_stem_ > –1.20 MPa (Padilla-Diaz et al., 2016), while for persimmon trees, the threshold was Ψ_stem_ > –0.80 MPa (Martinez-Gimeno et al., 2017). Our findings showed that State I for greenhouse tomato and celery indicated no stress or mild water stress, corresponding to soil moisture ranges of > 20% and > 19%, respectively. Meanwhile, State II indicated severe water stress, with soil moisture ranges of< 18% and< 16%, respectively. Previous studies on drip irrigation for these crops suggested maintaining soil moisture at 70% θ_f_ for optimal irrigation benefits. This corresponded to SWC ranging from 21.60% to 26.81% in tomato (Li et al., 2022b; Liu et al., 2009; Wang and Zheng, 2016) and 17.64% (loamy soil) to 29.90% in celery (Hartz, 2000; Yang et al., 2016). The analytical results in tomato conformed to the State I threshold range; however, celery results showed discrepancies. This discrepancy could be attributed to the varying soil water-holding capacities and irrigation frequencies across the experimental sites, resulting in a broad spectrum of SWC values in celery (Breschini and Hartz, 2002). Moreover, in this experiment, both plant species exhibited P_p_ states within the intermediate SWC range of 18%–20% (tomato) and 16%–19% (celery). In view of the intricate and changing real scenario, it is essential to conduct more sophisticated studies on water deficiency levels in the future and constantly modify the irrigation thresholds.

### 4.2 Evaluation of predictive models for leaf turgor in drip-irrigated tomato and celery

To determine the most appropriate model for predicting leaf turgor pressure, this study evaluated and compared the simulation accuracies of models built with various parameter combinations and machine learning techniques. According to the data, Combination 4 (substate P_p_ prediction based on meteorological factors and SWC) achieved the highest prediction accuracy, whereas Combination 1 (whole state P_p_ prediction based on meteorological factors) exhibited the lowest accuracy. This finding highlights that utilizing substate P_p_ and SWC as inputs significantly enhances the predictive capacity of these techniques by providing critical insights into plant water status. Notably, all three machine learning techniques (RF, XGBoost, and SVM) employed in this study demonstrated the capacity to replicate the dynamics of the P_p_ time series. Among them, RF outperformed XGBoost and SVM. This superiority could be attributed to the RF’s robust capacity to effectively manage high-dimensional data, its strong tolerance for noise and outliers, and its ability to reduce overfitting through ensemble learning (Janiesch et al., 2021). Studies by Shi et al. (2022) and Amir et al. (2021) indicated that these characteristics of RF enhance its feasibility and accuracy in simulating plant water physiology metrics. Furthermore, the RF can successfully capture nonlinear connections in the data since it is based on decision tree building principles (Djiemon et al., 2019). As a result, RF proved more adept at representing the complex nonlinear interactions between environmental factors and P_p_ in this investigation, including those related to different water stress states.

Concurrently, the feature significance evaluation of RF revealed that SWC was the most important characteristic (**Supplementary Figure S1**) highlighting its critical role in regulating P_p_ in greenhouse tomato and celery. To investigate the sensitivity of P_p_ to SWC dynamics under consistent meteorological conditions, scenarios were simulated where SWC was hypothetically increased or decreased by 5%, and the corresponding P_p_ was then predicted (**Supplementary Figure S2**). The initial SWCs were 20.93% (State I) and 17.06% (State II) for tomato, and 20.65% (State I) and 14.86% (State II) for celery. When SWC was increased by 5%, the State I P_p,max_ of tomato and celery decreased by 14.63 and 2.67 kPa, respectively, and the P_p_ curves in State II shifted toward State I. Conversely, the P_p_ value of tomato State II increased considerably. When SWC was decreased by 5%, the State I P_p,max_ of tomato and celery increased by 36.86 and 8.53 kPa, respectively, and the P_p_ signal of State II collapsed. This sensitivity analysis provides insights into irrigation trigger conditions and facilitates the analysis of irrigation effects. The data also offer a valuable reference for estimating a reasonable irrigation quota for greenhouse crops. Current predictive models for plant leaf turgor pressure often have limited consideration for soil moisture variability and tend to rely on simpler relationships with meteorological factors (Bader et al., 2014; Kant et al., 2014) or other plant water metrics (Barriga et al., 2022; Fernandes et al., 2017; Camoglu et al., 2021). While linear or single-metric approaches offer insights into plant water status, our integrated modeling approach significantly improves P_p_ prediction, particularly in response to dynamic environmental shifts. The integration of SWC into our advanced machine learning model enabled high predictive accuracy (R² > 0.958), demonstrating a robust capacity to capture complex environmental influences on P_p_. Thus, our model offers a more integrated and precise simulation of plant water dynamics, providing enhanced utility for fine-tuning irrigation strategies.

The prediction model proposed in this study demonstrates the capacity to forecast leaf turgor pressure based on meteorological and soil moisture variations. This model can also fill missing data for daily monitoring of turgor pressure using the features of RF (Emmanuel et al., 2021; Saini and Nagpal, 2023). In addition, the model exhibits substantial economic efficiency. Egea et al. (2017) calculated the equivalent interannual cost of precision irrigation management using the LPCP probe for commercial olive orchards to be 204 €·year^-1^·ha^-1^. Despite being significantly less expensive (61.58% and 53.64% lower, respectively) than trunk diameter and sap flow probes, this interannual cost was still unaffordable for the majority of users. The proposed model might optimize resource allocation by reducing the need for monitoring probes, thereby lowering overall costs. However, the model also has certain limitations. For example, it fails to account for the spatial and temporal heterogeneities of meteorological conditions and soil moisture levels across various locations within the greenhouses. Future projections of leaf turgor pressure at the global geographic scale will need to take into account microenvironmental differences. Furthermore, the dataset of the model is limited to State I and State II data from 2 years of greenhouse tomato and celery drip irrigation trials. It is necessary to further augment the database to broaden the model’s applicability.

## 5 Conclusions

The presented study found that diurnal P_p_ curves of drip-irrigated greenhouse tomato and celery exhibited two distinct states across growth stages: a unimodal pattern (State I) and a troughed pattern (State II), with significant differences among the treatments (*P* < 0.05). State I corresponded to no or mild water stress, defined by SWC thresholds above 20% for tomato and above 19% for celery. Conversely, State II represented severe stress, occurring at SWC below 18% for tomato and below 16% for celery. Compared to full irrigation, non-full irrigation significantly increased the incidence of State II and resulted in higher values of P_p,max_ and P_p,min_ in both tomato (increase of 15.39%–138.39%) and celery (increase of 3.44%–94.02%). Analysis of State I revealed that solar radiation was the most favorable factor influencing P_p_. In contrast, SWC exerted a substantial negative influence, meaning that P_p,max_ and P_p,min_ increased as SWC decreased within this state. The correlations between environmental factors and P_p_ were generally weaker in State II. Subsequent to the abovementioned analysis, a Random Forest-based prediction model was developed to estimate the change in leaf turgor pressure of greenhouse tomato and celery. The model utilized the meteorological factors and SWC as inputs and predicted P_p_ values for both states. This approach yielded the smallest prediction error and the highest accuracy (R^2^ = 0.995). Future work should focus on expanding the application range and prediction scale of the model to further support precision irrigation management in greenhouses.

## Data Availability

The original contributions presented in the study are included in the article/**Supplementary Material**. Further inquiries can be directed to the corresponding author.
