# Supplementary material for: Water status diagnosis in greenhouse drip-irrigated tomato and celery using leaf turgor dynamics and machine learning
Source: Front Plant Sci. 2026 Jan 16;16:1743809. doi: 10.3389/fpls.2025.1743809 (PMC12855137; doi:10.3389/fpls.2025.1743809)
Supplement: Supplementary file 1 [file SupplementaryFile1.docx]

**Table S1** Average soil physicochemical parameters in the 0-30 cm soil layer during tomato fruit development under TB and T0 treatments in 2021 and 2022. * indicates P < 0.05. ** indicates P < 0.01. Both highlight the differences in treatments for the same year.

| Year | Treatment | Soil water content/% | Bulk density/g·cm^-3^ | Total porosity/% | pH | Redox potential/mV |
| --- | --- | --- | --- | --- | --- | --- |
| 2021 | TB | 20.36±0.11** | 1.31±0.02** | 50.41±0.68** | 8.58±0.06 | 581.34±4.88 |
|  | T0 | 18.52±0.12** | 1.41±0.02** | 46.62±0.71** | 8.44±0.05 | 569.31±4.59 |
| 2022 | TB | 20.28±0.16** | 1.27±0.01** | 51.94±0.39** | 8.61±0.03* | 617.30±7.06 |
|  | T0 | 18.90±0.24** | 1.35±0.01** | 49.08±0.41** | 8.49±0.03* | 604.86±8.74 |

**Table S2** Growth stage division for monitoring leaf turgor pressure in tomato and celery drip irrigation systems.

| Year | Crop | Growth stage | Time frame | Duration/d |
| --- | --- | --- | --- | --- |
| 2021-2022 | Tomato | Pre-fruiting stage | 2021.07.13-2021.08.04 | 23 |
|  |  | Mid-fruiting stage | 2021.08.05-2021.08.25 | 21 |
|  |  | Late-fruiting stage | 2021.08.26-2021.09.23 | 29 |
|  | Celery | Outer leaf growing stage | 2021.11.18-2021.12.10 | 23 |
|  |  | Standing stage | 2021.12.11-2021.12.31 | 21 |
|  |  | Heartleaf growing stage | 2022.01.01-2022.01.20 | 20 |
| 2022-2023 | Tomato | Pre-fruiting stage | 2022.07.22-2022.8.13 | 23 |
|  |  | Mid-fruiting stage | 2022.08.14-2022.09.04 | 22 |
|  |  | Late-fruiting stage | 2022.09.05-2022.10.01 | 27 |
|  | Celery | Outer leaf growing stage | 2022.12.02-2022.12.25 | 24 |
|  |  | Standing stage | 2022.12.26-2023.1.15 | 21 |
|  |  | Heartleaf growing stage | 2023.01.16-2023.02.12 | 28 |


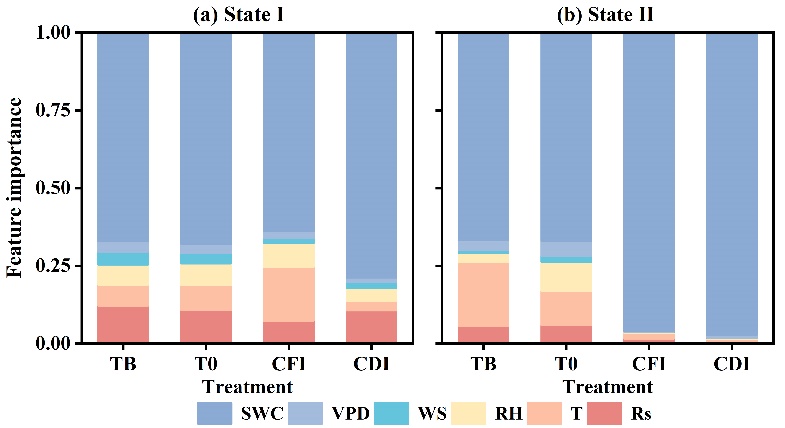


**Figure S1** Feature importance of the Combination 4 with RF State I (a) and State II (b).


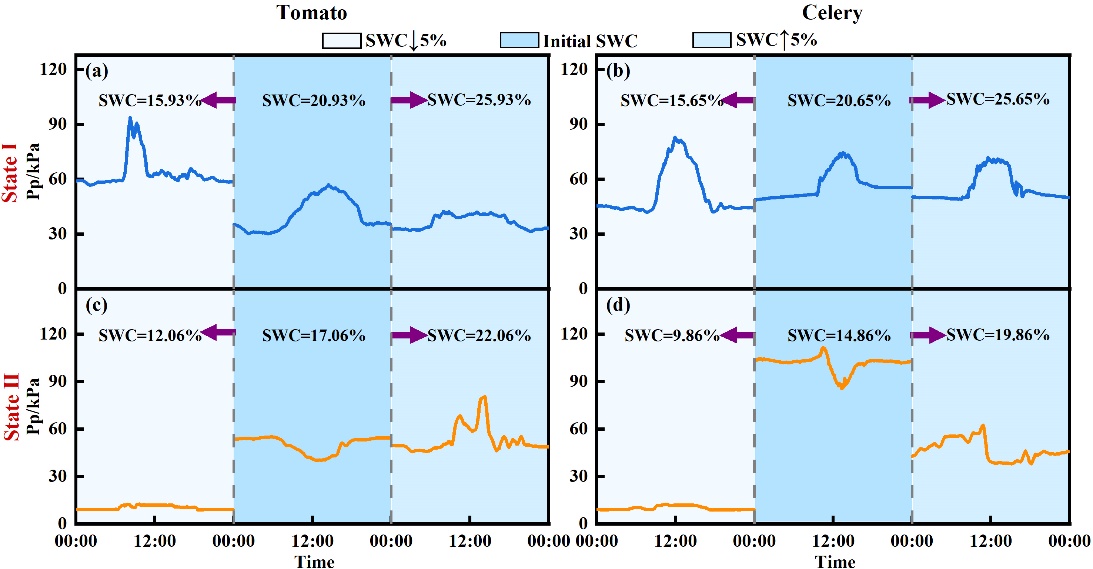


**Figure S2** Machine learning model-based P_p_ predictions for drip-irrigated tomato (a and c) and celery (b and d) State I and State II at 5% increase/decrease in the SWC.
